# Supplementary figures and images for: Climbing the Giara: A quantitative reassessment of movement and visibility in the Nuragic landscape of the Gesturi plateau (South-Central Sardinia, Italy)
Source: PLoS One. 2023 Aug 3;18(8):e0289023. doi: 10.1371/journal.pone.0289023 (PMC10399907; doi:10.1371/journal.pone.0289023)

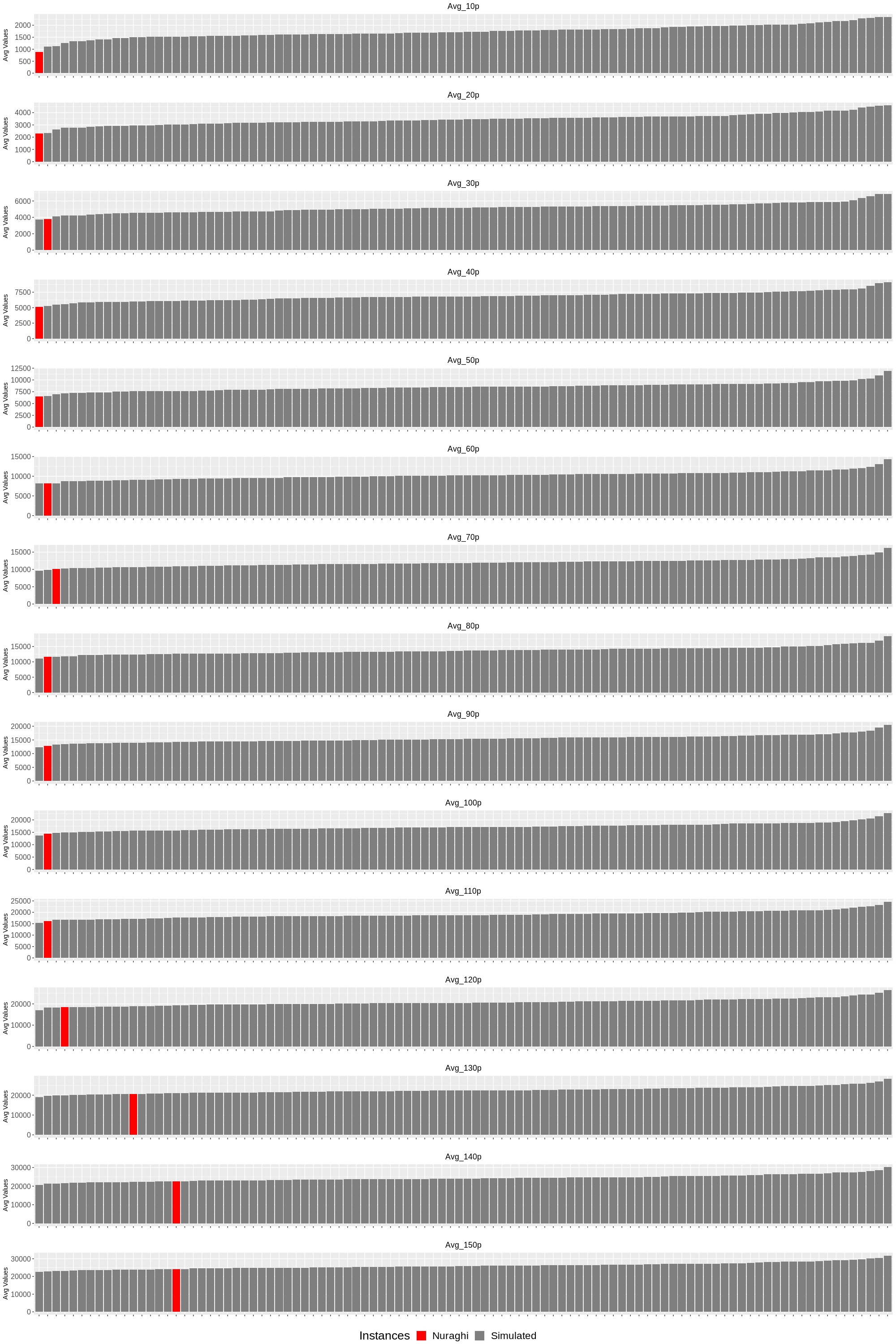

Supplement: S1 Fig — (TIF) [file pone.0289023.s005.tif]

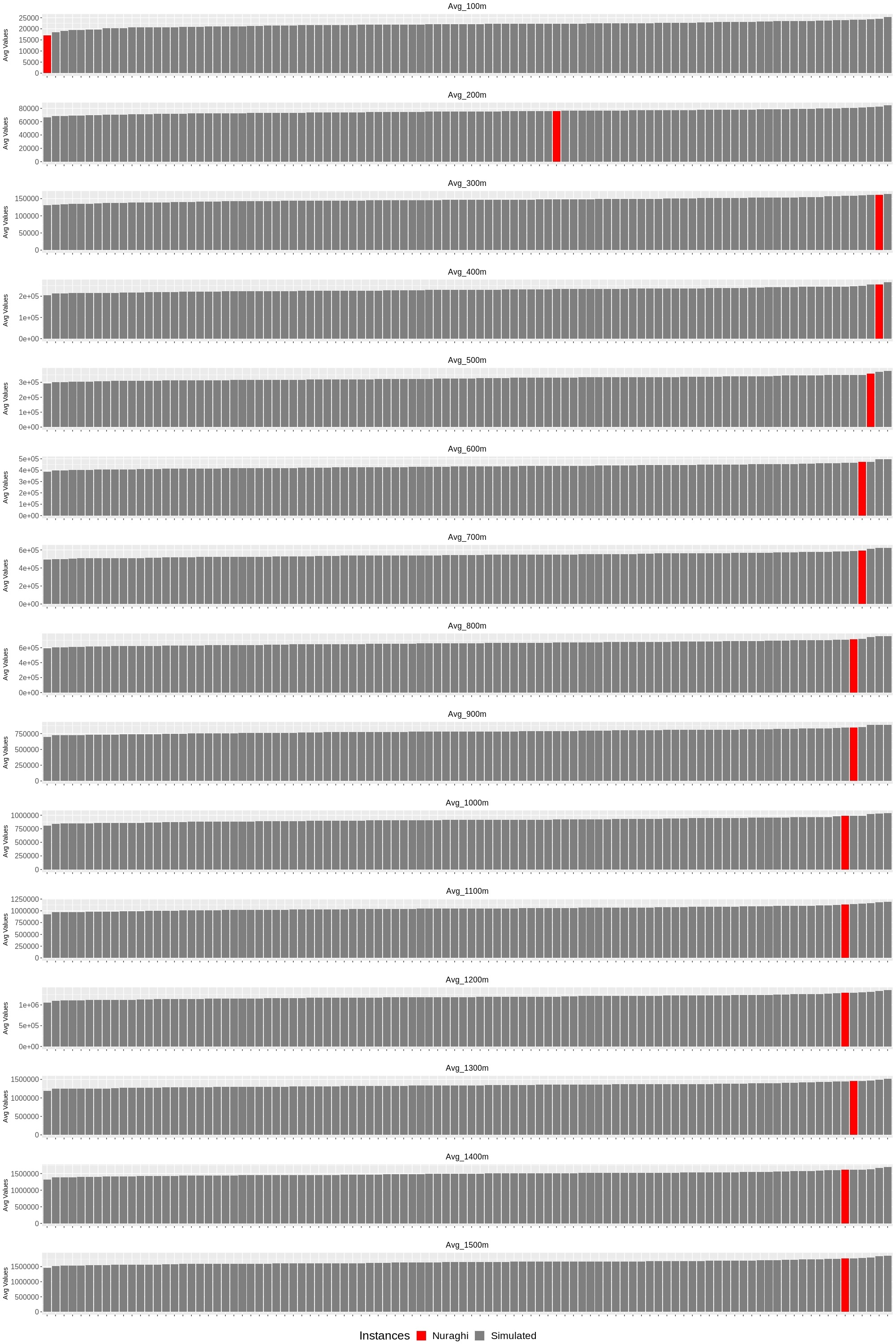

Supplement: S2 Fig — Buffer radiuses of 100 m to 1500 m. (TIF) [file pone.0289023.s006.tif]

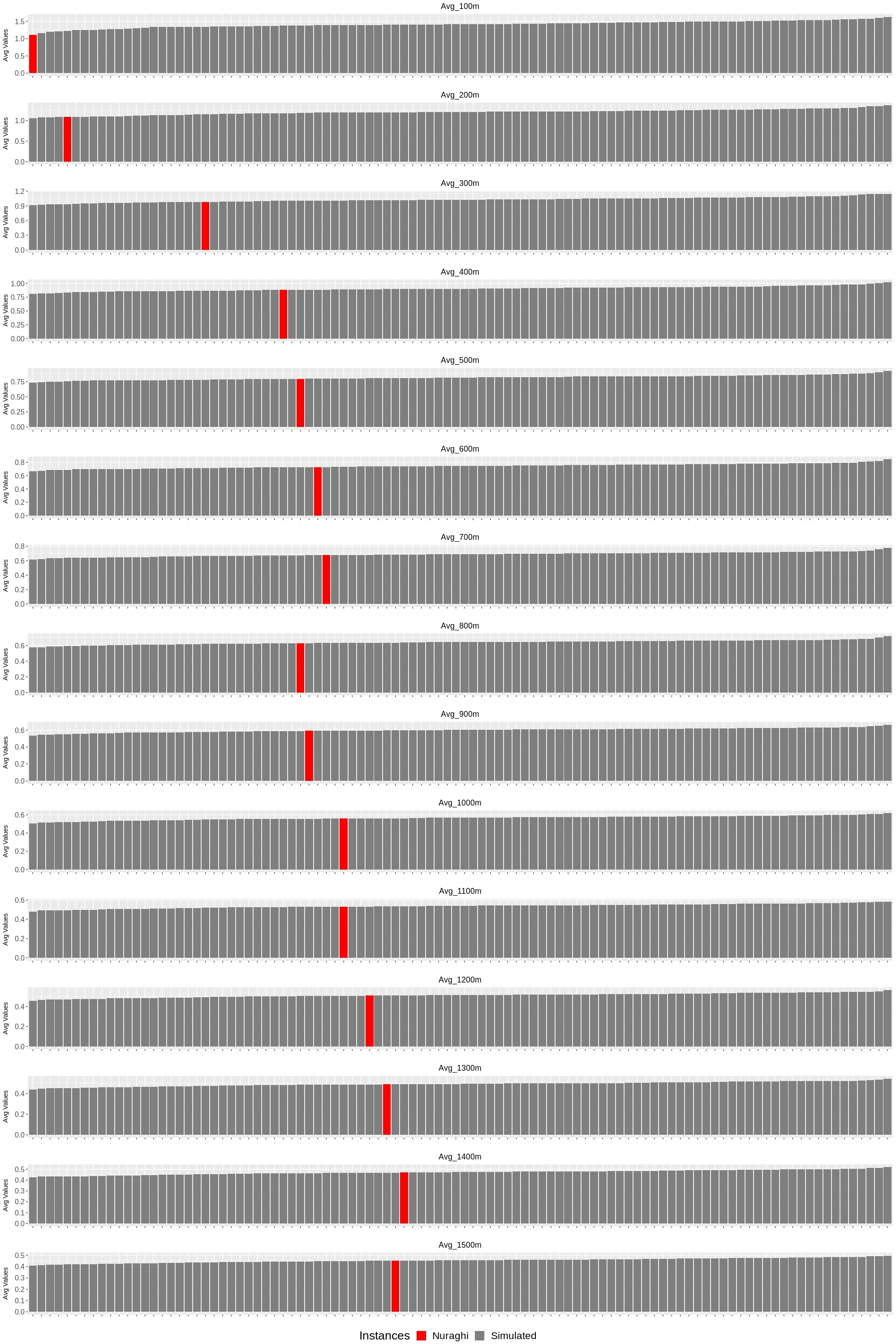

Supplement: S3 Fig — Buffer radiuses of 100 m to 1500 m. (TIF) [file pone.0289023.s007.tif]

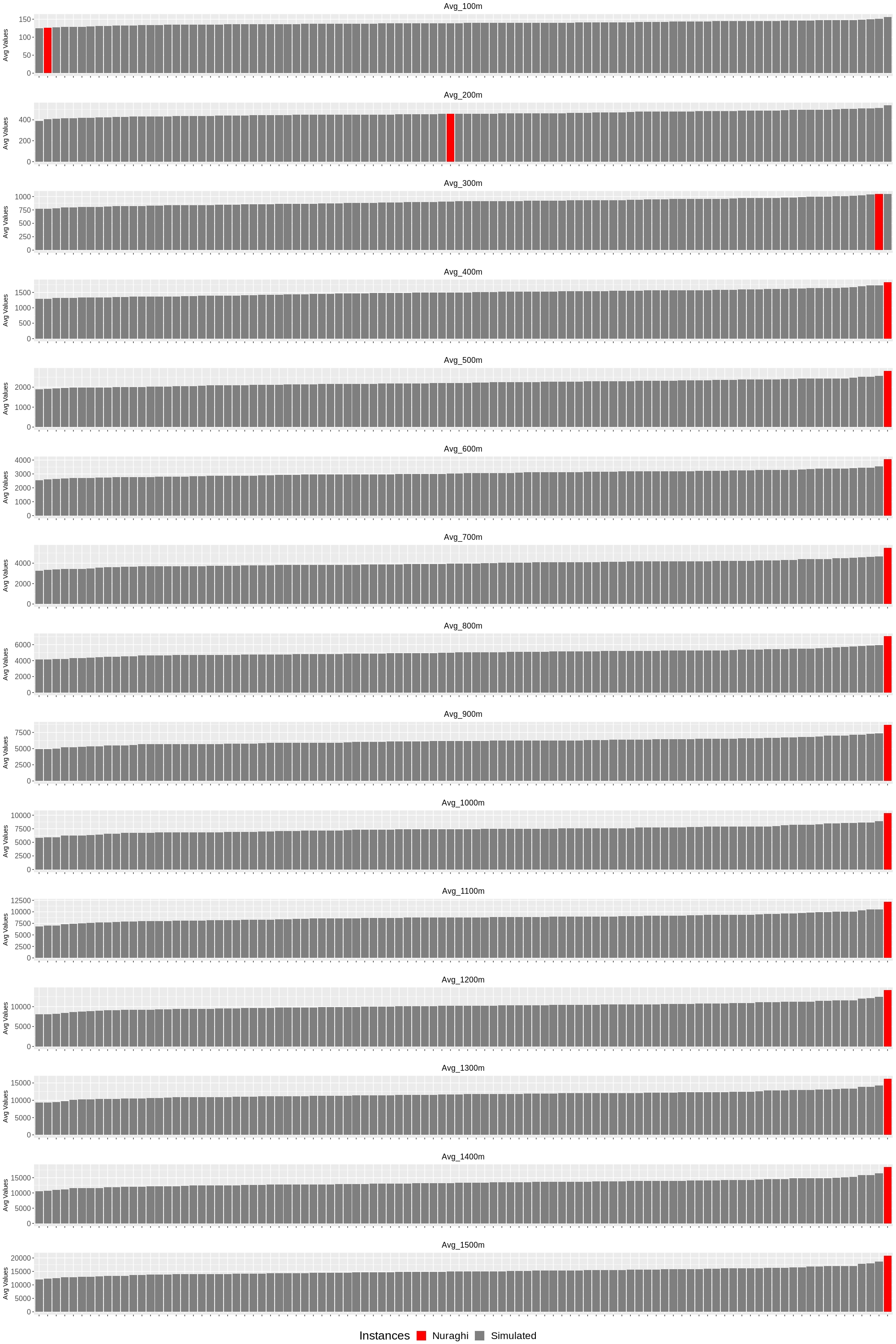

Supplement: S4 Fig — Buffer radiuses of 100 m to 1500 m. (TIF) [file pone.0289023.s008.tif]

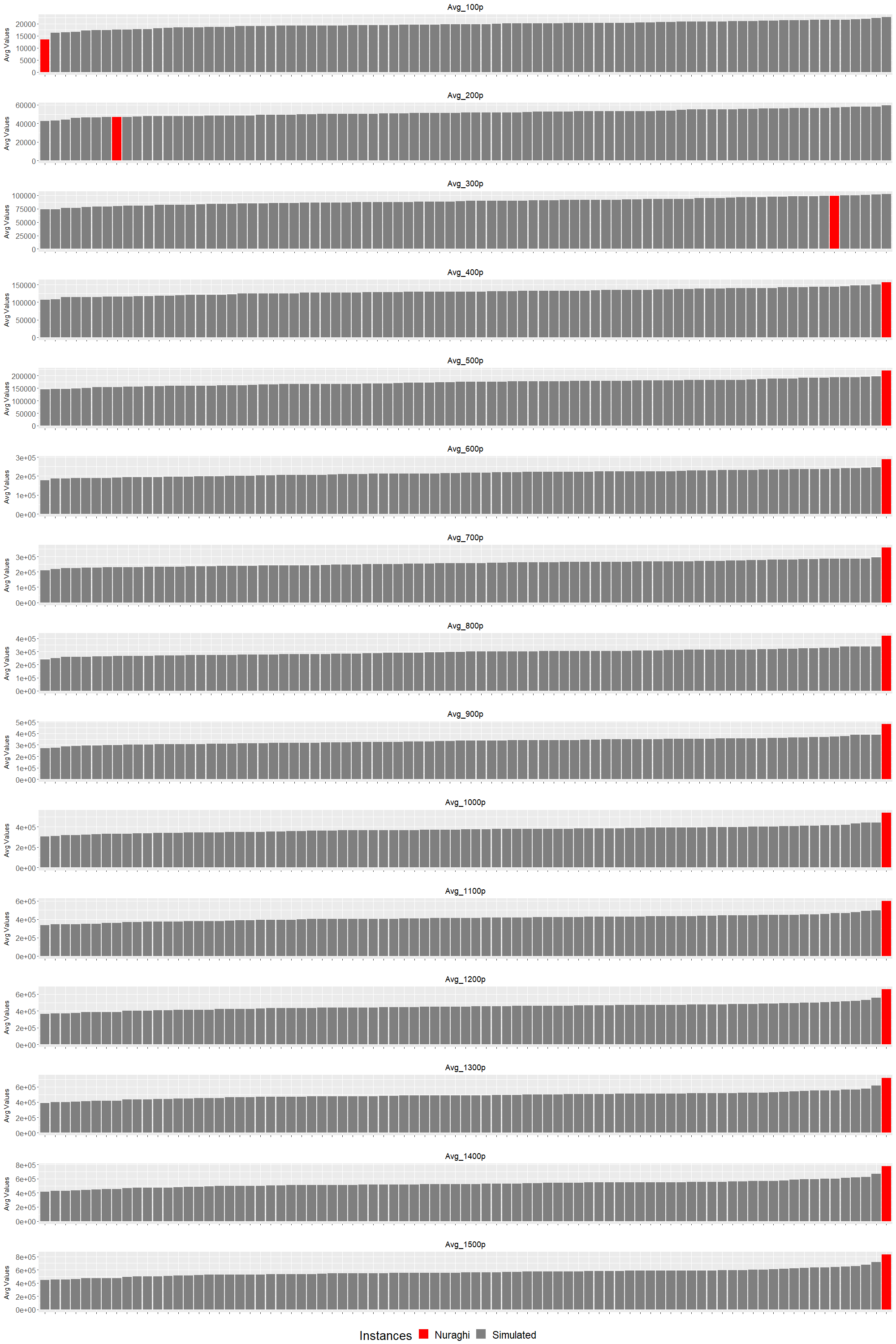

Supplement: S5 Fig — Buffer radiuses of 100 m to 1500 m. (TIF) [file pone.0289023.s009.tif]

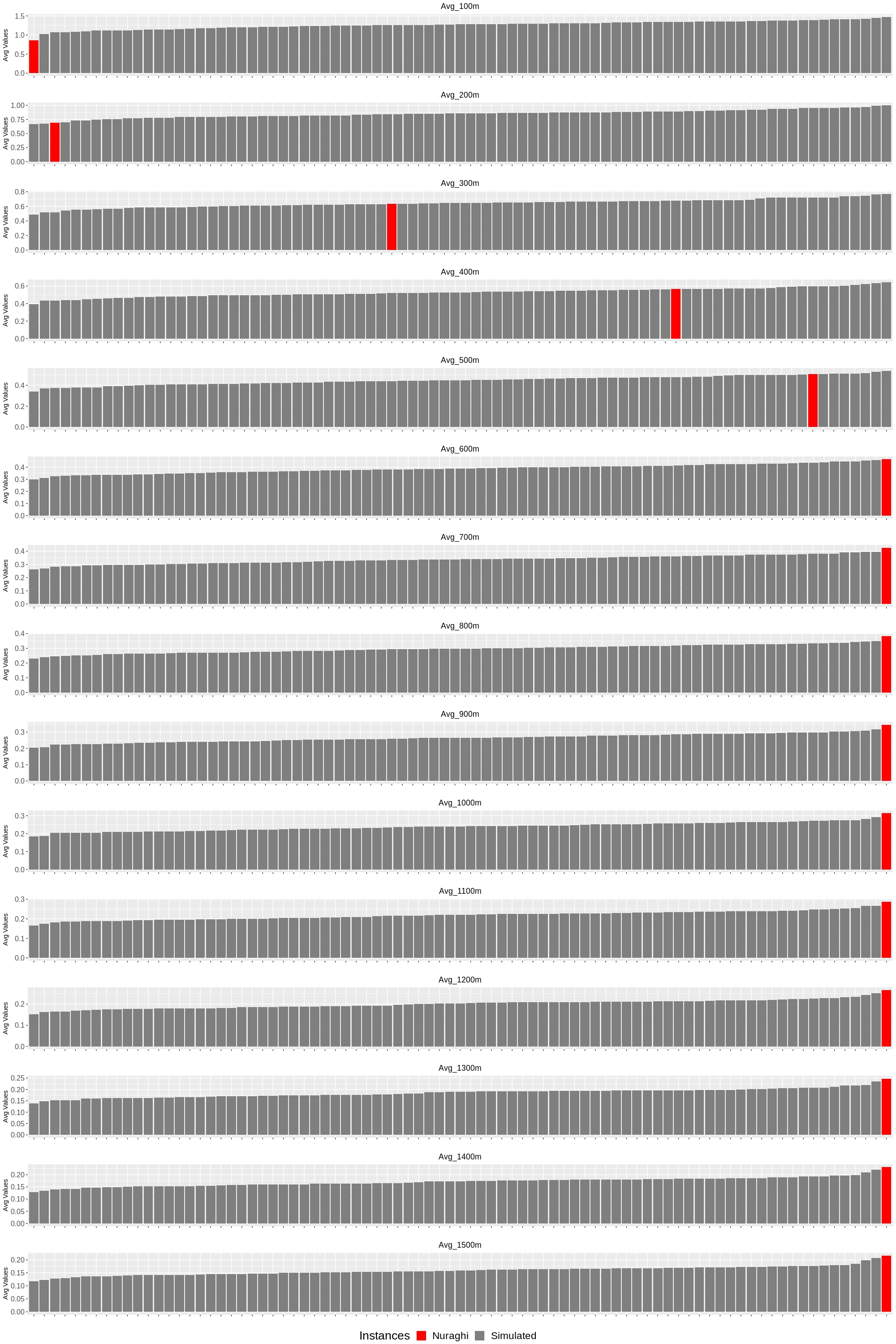

Supplement: S6 Fig — Buffer radiuses of 100 m to 1500 m. (TIF) [file pone.0289023.s010.tif]
